# Psychometric properties of the portuguese version of the chronic urticaria quality of life questionnaire (CU-Q_2_oL)

**DOI:** 10.1186/s12955-019-1266-y

**Published:** 2019-12-30

**Authors:** Pedro Lopes Ferreira, Margarida Gonçalo, José Alberto Ferreira, Ana Célia Costa, Ana Todo-Bom, Cristina Lopes Abreu, Ana Rita Travassos, Pedro Andrade, Ilaria Baiardini, Giorgio Walter Canonica

**Affiliations:** 1 Centre for Health Studies and Research and Faculty of Economics, University of Coimbra, Coimbra, Portugal; 2 Dermatology Department, University Hospital and Faculty of Medicine, University of Coimbra, Coimbra, Portugal; 3 Immuno-allergology Department, Vila Nova de Gaia / Espinho Hospital Centre, Vila Nova de Gaia, Portugal; 4 Immuno-allergology Department, Lisbon-North Hospital Centre, Lisbon, Portugal; 5 Immuno-allergology Department, University Hospital and Faculty of Medicine, University of Coimbra, Coimbra, Portugal; 6 Immuno-allergology Department, Matosinhos Health Local Unit, Matosinhos, Portugal; 7 Dermatology Department, Lisbon-North Hospital Centre, Lisbon, Portugal; 8 Dermatology Department, Matosinhos Health Local Unit, Matosinhos, Portugal; 9 Department of Biomedical Sciences, Humanitas University, Milan, Italy; 10 Department of Biomedical Sciences, Humanitas University, Milan, Italy; 11 Personalized Medicine, Asthma and Allergy Clinic, Humanitas Research Hospital, Milan, Italy

**Keywords:** Patient Reported Outcome Measures, Quality of life, Reproducibility of results, Chronic urticaria

## Abstract

**Background:**

Chronic urticaria is defined as the appearance of urticarial lesions and/or angioedema during a period of more than six weeks. We aimed at developing the Portuguese version of the Chronic Urticaria Quality of Life Questionnaire (CU-Q_2_oL) and at testing its reliability and the content, construct and criterion validity.

**Methods:**

The forward-backward approach to a linguistic equivalence was followed, after which a clinical review and a cognitive debriefing with patients were performed. The intraclass correlation coefficient checked test-retest reliability with patients filling the same questionnaire with one week apart and the Cronbach’s alpha indicator assessed the internal consistency. Construct validity was tested by an exploratory factor analysis and by hypothesis tests involving sociodemographic and clinical patient characteristics, including the urticaria control test (UCT). On the other hand, criterion validity was tested through correlations with the Short-Form Health Survey SF-36, EQ-5D-5 L, and the Dermatology Quality of Life Index (DLQI).

**Results:**

A total of 162 patients from seven hospital units were included. The mean (standard deviation) age was 42.6 (13.3) and 81.6% were female. CU-Q_2_oL was entirely filled by all respondents. Internal consistency was 0.947 for the overall score, ranging from 0.661 (limits) to 0.899 (sleep problems) and the corresponding reproducibility indicator was 0.910, based on 23 patients and ranging from 0.711 (swelling) and 0.957 (looks). Exploratory factor analysis in general confirmed the original structure originally obtained by the authors. All CU-Q_2_oL dimensions were highly correlated with DLQI Index and differentiated well between males and females, and between different levels of wheals and pruritus. In addition, moderate negative correlations were found between Cu-Q_2_oL scores and the dimensions from SF-36 and EQ-5D-5 L.

**Conclusions:**

The satisfactory metric properties confirmed the cultural adaptation and validity of CU-Q_2_oL into Portuguese population, providing the clinicians with a valid tool to evaluate the impact of chronic urticaria on patient’s QoL and therefore adjust their treatment.

**Trial registration number:**

Not applicable.

## Background

Every chronic illness produces a significant impact on patients’ life, affecting both physical and psychosocial domains. Chronic urticaria (CU) can be defined as the appearance of urticarial lesions (wheals) and/or angioedema during a period of more than six weeks [1–5]. There are major problems regarding etiology, pathogenic mechanisms and pharmacological treatment of CU and its management ultimately aims at preserving the well-being and the quality of life of patients.

In fact, chronic urticaria can lead to changes in sleep patterns, daytime sleepiness, ability to concentrate, altered perception of self-image, social isolation, psychological changes and even anxiety and depression [6]. It also yields relevant direct and indirect costs, including drugs, medical appointments, emergency visits, hospitalization, absenteeism and presenteeism [7].

Few studies had addressed quality of life (QoL) in patients with chronic urticaria until 2005, when Baiardini et al. created the Chronic Urticaria Quality of Life Questionnaire (CU-Q_2_oL) [1], an Italian measure specifically designed for CU patients to express their perspectives. Until then the QoL of patients with CU was only measured through generic health status instruments such as the Short-Form Health Survey SF-36 [8] or the Nottingham Health Profile [9].

Although generic instruments can be used in all diseases and populations, enabling an easier comparison of the impact of health states associated with various clinical conditions, they do not measure the precise burden of a particular disease, and they may not be enough sensitive to changes in health status. Specific instruments, on the other hand, are more sensitive to small differences in health and changes overtime, showing higher face validity, thus better supporting therapeutic decisions. For instance, the dimension ‘looks’ is never addressed by generic instruments, in spite of being very meaningful for patients with chronic urticaria. Therefore, the cultural adaptation of the CU-Q_2_oL to the Portuguese reality was somehow urgent in order to know the effective impact of CU in patients and therefore allow providers and researchers to compare their results with those obtained in different countries and to participate in international studies.

There are no specific data for Portugal regarding the incidence and/or prevalence of chronic urticaria but, based on international data, it is estimated that CU affects up to 1% of the Portuguese population at any given time. Two thirds represent forms of chronic spontaneous urticaria [6, 10, 11], which can have a high impact on consumption of healthcare resources and direct and indirect costs. According to the single study concerning costs in Portugal, which is on agreement with international data, the average cost to treat a patient with severe chronic spontaneous urticaria is estimated to be 6234€ over five years, of which 4220€ correspond to National Health Service direct costs and the remaining 2014€ to out-of-pocket indirect costs [12]. A systematic implementation of a valid and reliable measure of quality of life may alert doctors and patients to a timely approach to the impact of the situation.

The aim of this study was to create a valid and reliable questionnaire in European Portuguese from the original CU-Q_2_oL, to measure the quality of life and the burden experienced by individuals with chronic urticaria. For this purpose, we (i) linguistically and culturally adapted the CU-Q_2_oL from Italian to Portuguese; (ii) assessed reliability, validity, interpretability and acceptance of the Portuguese version; and (iii) estimated the health-related quality life of a sample of patients with chronic urticaria.

## Methods

### Linguistic and cultural adaptation

We followed the internationally defined methodology for the linguistic and cultural adaptation for the validation of outcome measures, as well as the author’s methodological proposal. Therefore, after the authors’ permission for the creation of a Portuguese version of the CU-Q_2_oL, we started with the translation of the Italian version to Portuguese and followed the recommendations proposed by the COSMIN checklist [13] and by the sequential approach [14].

This phase included the elaboration of two local independent versions from two experienced bilingual Portuguese translators who, based on the original Italian questionnaire preformed forward translations into Portuguese. Both translations were merged in a reconciled version drawn up between the translators and the study team. Another local translator, bilingual Italian native speaker, a researcher in the University of Coimbra, who did not have any access to the original Italian version of the questionnaire, then back translated this merged version. This back-translation was finally compared with the original version to guarantee a semantic equivalence.

We also asked a clinician to perform a scientific review of the final translation. This allergy hospital specialist was asked to look at both versions and comment. Three alternatives of answers were given to her: (i) if happy with the translation, it was only required to use a check sign to say it in the “comments section”; (ii) if terms should have substantial corrections to add, she should give a new proposal in the section “suggestions proposed”; and (iii) if assumed that there is more than one correct form to translate the sentence, she should include her suggestion on the section “possible alternative for further cognitive test”.

Next, we ran a cognitive debriefing meeting with 10 chronic urticarial patients. Our aim was to detect whether the Portuguese version we had was acceptable by patients and whether its contents was understandable, no ambiguous, no redundant and easy to fill. This sample of actual patients approximately respected the age-gender distribution of the target patient group, with only one exception: we forced to have patients from the lowest possible education level, as we assumed that if interpretation problems didn’t occur with this type of patients they won’t occur with patients with higher education level.

At this phase, our main goal was to obtain a conceptual and linguistic version, equivalent to the Italian original. That is, a Portuguese version where the items would have the same meaning and content as the corresponding items in the original version.

### Study design

This was an observational multicenter study aimed at validating a diagnostic scale and analyzing the health-related quality of life of a sample of patients. Dermatologists and imuno allergologists from seven dermatology and imuno-allergology departments of four Portuguese public hospitals from the areas of Lisbon, Coimbra and Oporto conducted the study during regular medical appointments. The National Data Protection Agency and the Ethics Committees of all hospitals approved the study and all participants gave their informed consent after previous information about the objective of this study, its benefits, potential risks and possible discomfort. There was, under no circumstances, any interference with the health professional decision regarding the best-suited medical approach to each patient.

### Participants

We included consecutive patients from the different units who (i) had at least 18 years of age, (ii) suffered from chronic urticaria for at least six months, (iii) had capacity to give consent to participate in the study, and (iv) knew how to read, write and understand Portuguese. Excluded were (i) unstable patients or with uncontrolled symptoms and perceived by clinicians as not having ability to fill the measures, (ii) cognitively affected patients, or (iii) who did not understand Portuguese.

After data collection, the information was registered in a digital device without any identification of patients. Data was analyzed and hypotheses were tested in order to demonstrate the reliability and validity of the Portuguese version of the CU-Q_2_oL.

### Measurement instruments

In this study, health-related quality of life was measured through specific and generic instruments. Specific instruments included the Portuguese version of the CU-Q_2_oL and the Dermatology Quality of Life Index (DLQI). Among the generic instruments we selected the generic health status measure SF-36 and the health preference quality of life EQ-5D-5 L. Sociodemographic and clinic data were also collected, including the measurement of the severity of the urticaria and its control by the Urticaria Control Test (UCT).

- CU-Q_2_oL is the first disease-specific instrument designed to measure the quality of life of patients with chronic urticaria [1]. The authors of the initial Italian version had the goal to create a self-administered, easy and fast answering measurement instrument to be filled without any assistance and to be able to capture the physical, psychosocial and practical aspects relevant to patients with chronic urticaria [ 1]. It was initially created by professionals and patients and consisted of 37 items with a recall period of two weeks; the items emerged from experts and researchers in immunology, dermatology and immuno-allergology, as well as a panel of 60 patients affected by CU. It was later reduced to 23 items corresponding to an overall impairment score and six dimensions: pruritus, swelling, impact on life activities, sleep problems, limits, and looks, as follows:

**Table:** 

**Pruritus**	**Sleep problems**	**Looks**
1 pruritus	11 ease of falling asleep	19 medication adverse effects
2 wheals	12 walking up during the night	20 bothersome
**Swelling**	13 daytime tiredness	21 embarrassing in public
3 eyes swelling	14 ability to concentrate	22 use of cosmetics
4 lips swelling	15 nervousness	23 choice of clothes
**Impact on life activities**	**Limits**	
5 work	16 bad mood	
6 physical activities	17 limits in choosing food	
7 quality of sleep	18 sport	
8 free time		
9 social relations		
10 eating		

Answering the questionnaire, patients express how troubled they are, by scoring each item on a 5-point Likert-type scale from 0 (not at all) to 4 (extremely). For each dimension, the corresponding items are summed to obtain a score, which is converted to a scale from 0 to 100 through a linear transformation. Similar procedure is applied to an overall score. Higher values always correspond to higher QoL impairment, which means worse QoL [1].

In its original version, this measure showed good values of convergent validity with SF-36, internal consistency with Cronbach alpha scores between 0.65 and 0.83, reliability with good Intraclass Correlation Coefficient (ICC) for four items and greater or equal to 0.75 for the other items and that is, standards of quality that ensure a good measuring tool to evaluate the burden in chronic urticaria patients. It has been translated and adapted in several languages, such as Brazilian-Portuguese [15], German [16], Greek [17], Israeri [18], Persian [19], Polish [20], Spanish [21], Thai [22], and Turkish [23].

- DLQI is a dermatology-specific questionnaire with 10 items [24] and assesses six different aspects that may affect the patients’ QoL: symptoms and feelings, daily activities, leisure activities, work or school, personal relationships, and treatment. Higher scores indicate a greater impairment in QoL [24]. Each of the 10 questions is scored from 0 (not at all) to 3 (very much) and the overall DLQI score is calculated by summing up the scores from each question, resulting in a numeric score between 0 and 30. Higher scores indicate a greater impairment in QoL. The impact of the DLQI scores on a patient’s life is as follows: 0 to 1 = no effect; 2 to 5 = small effect; 6 to 10 = moderate effect; 11 to 20 = very large effect; 21 to 30 = extremely large effect.
- SF-36, with its 36 items, measures eight major health dimensions, all of them on a scale of 0–100, with the extreme anchors corresponding, respectively, to death and perfect health status [25, 26]. The effectiveness dimensions are physical function, role limitations due to physical or emotional problems, intensity and discomfort caused by pain, general health, vitality, social function, and mental health. Higher scores mean a better perceived health status.
- EQ-5D-5 L is a generic QoL instrument consisting of five dimensions of health (mobility, self-care, usual activities, pain/discomfort, and anxiety/depression) and a visual analogue scale (VAS) for rating health on the precise day. Weighted scoring produces an EQ-5D-5 L index score [27]. The EuroQoL Group has approved the Portuguese version as well as the corresponding value set [28].
- UCT is a patient-reported outcome instrument to retrospectively assess urticaria control [29]. Each item has five answer options (scored with 0 to 4 points). Low scores indicate high disease activity and low disease control. Accordingly, the minimum and maximum UCT scores are, respectively, 0 and 16, with 16 points indicating complete disease control.

The order of administration for the instruments was the following: first, we asked patients for clinical information regarding his/her chronic urticaria, before addressing their assessment of the urticaria control (UCT); next, we applied the preference quality of life measure EQ-5D-5 L, followed by the DLQI, the CU-Q_2_oL and the SF-36; at last, we asked for sociodemographic variables.

### Reliability

To address the reliability, we tested the intertemporal stability and the internal consistency. The former was tested using the ICC in a 1-week test-retest design with no clinical intervention during this week. A score smaller that 0.5 is considered weak, between 0.5 and 0.75 moderate, between 0.75 and 0.9 good, and larger than 0.9 excellent [30].

On the other hand, the internal consistency, representing the homogeneity among the individual items, was tested using the Cronbach’s alpha coefficient, which should have scores between 0.7 and 0.9 [31].

The following two hypotheses were formulated:

H1: The Portuguese version of the CU-Q_2_oL shows good internal consistency.

H2: The Portuguese version of the CU-Q_2_oL shows good intertemporal stability.

### Validity

For a measure to be precise, it is essential that it measures/evaluates what it is supposed to measure. In what concerns the validity tests, we addressed the three strands of content, construct, and criterion [31, 32]. The content validity, measuring the relevance of the items, has already been tested through the cognitive interviews of 10 patients and with the reviews performed by clinicians during the linguistic and cultural adaptation phase.

The construct validity addresses the ability of the instrument to measure theoretical concepts. Following some authors [31, 32], we used the two major ways to test the construct validity: structural validity and hypothesis testing. The structural validity was tested by using exploratory factor analyses. Hypotheses testing assumes the formulation of several hypotheses with known groups stratified by sociodemographic variables (sex, age, family status, employment status and education level) and some clinical variables (disease duration, angioedema, type and severity of urticaria, comorbidities, type of treatment, and control of urticaria). Student t-test and ANOVA were used to test the CU-Q_2_oL scores differences on these known groups.

The following three hypotheses were formulated:

H_3_: Exploratory factor analysis replicates the original structure of CU-Q_2_oL.

H_4_: CU-Q_2_oL is able to discriminate based on sociodemographic variables.

H_5_: CU-Q_2_oL is able to discriminate based on clinical variables.

To test the criterion validity we used bivariate statistical analyses (Pearson’s r correlation coefficients) between the dimensions of the Portuguese version of the CU-Q_2_oL and other measuring instruments. Correlations less than 0.3, between 0.3 and 0.5, and higher than 0.5 were defined as weak, moderate and strong, respectively [33].

These other instruments included the generic health status instrument SF-36, the generic quality of life instrument EuroQoL EQ-5D-5 L and the dermatology-specific instrument DLQI. We expected to evidence the similarities and differences between measured concepts.

The following three hypotheses were formulated:

H_6_: CU-Q_2_oL dimensions are correlated with the SF-36 dimensions.

H_7_: CU-Q_2_oL dimensions are correlated with the EQ-5D-5 L.

H_8_: CU-Q_2_oL dimensions are correlated with the DLQI dimensions.

Taking into account that SF-36 is a generic health status measure, a priori we do not expect to have large correlations with CU-Q_2_oL. On the other hand, some significant correlations are expected with both EQ-5D-5 L index and VAS. Finally, because DLQI is a dermatology-specific questionnaire we expect to have large correlations with CU-Q_2_oL.

### Statistical analysis

Floor and ceiling effects were checked on overall CU-Q_2_ol score and dimensions. These effects exist whenever more than 15% of the respondents lie, respectively, in the lowest and the highest possible score [31, 32].

## Results

### Linguistic and cultural adaptation

During the forward-backward process, no major differences were found and, due to the layout of this questionnaire, some minor changes were produced in the Portuguese version after the comparison between the backward version and the original. Clinical review also yielded to minor changes in the Portuguese version. On the other hand, on the cognitive debriefing meeting, no understandability, ambiguity or redundancy errors were mentioned. Only the wording of some questions had to be changed in order to have a more colloquial questions. At the end of this process, a complete report was send to and approved by the authors of CU-Q_2_oL.

### Sample and reliability

A total of 162 patients from seven units were included in this study. Table 1 presents the main sociodemographic and clinical characteristics.

**Table 1:** Sample demographic and clinic characteristics (*n* = 162)

Variable	Value	No	%
Gender	Male	29	18.4
	Female	129	81.6
Age (years)	< 25	45	28.7
	[25–45[	44	28.0
	≥ 45	68	43.3
	Minimum – Maximum	18–79	
	Mean ± Standard deviation	42.6 ± 13.3	
Marital status	Single	39	24.8
	Married	99	63.1
	Widowed/divorced/separated	19	12.1
Years of education	0–4	19	12.3
	5–9	54	34.8
	10–12	36	23.2
	> 12	46	29.7
Employment status	Employed	110	70.1
	Retired	13	8.3
	Unemployed	18	11.5
	Other	16	10.2
Duration of urticaria (years)	Minimum – Maximum	0–49	
	Mean ± Standard deviation	5.6 ± 7.2	
Angioedema	Yes	70	47.0
	No	79	53.0
Type of urticaria	Chronic spontaneous urticaria	135	83.3
	Physical urticaria – pressure	32	19.8
	Physical urticaria – cold contact	7	4.3
	Physical urticaria – dermographic	18	11.1
	Physical urticaria – other	13	8.0
Pruritus level (UAS7)	None	30	19.0
	Light	53	33.5
	Moderate	53	33.5
	Intense	22	13.9
Number of wheals (last week) (UAS7)	None	50	32.7
	< 20	54	35.3
	[20–50]	39	25.5
	> 50	10	6.5
Comorbidities	Allergic rhinitis	43	26.5
	Asthma	20	12.3
	Drug allergies	32	19.8
	Food allergies	17	10.5
	Atopic dermatitis	7	4.3
	Contact dermatitis	10	6.2
	Diabetes mellitus	12	7.4
	Thyroid disorders	28	17.3
	Peptic ulcer	6	3.7
	Depression	23	14.2
	Others	26	16.0
Urticaria treatment	None	7	4.3
	Anti-histaminic once a day	52	32.1
	Anti-histaminic more than once a day	80	49.4
	Omalizumab	37	22.8
	Other	21	13.0
Urticaria control (UCT)	Poorly controlled (UCT < 12)	134	82.7
	Well controlled (UCT ≥ 12)	28	17.3
	Minimum – Maximum	1–14	
	Mean ± Standard deviation	8.2 ± 2.8	

Mean (standard deviation) age was 42.6 (13.3), 81.6% were female, 63.1% were married, 70.1% were employed, and almost 35% had 5 to 9 years of education. These patients suffered from the disease in average for the last 5.6 years (median of 3 years). The main diagnosis was chronic spontaneous urticaria (83.3%) and the most frequent comorbidities were allergic rhinitis (26.5%), drug allergies (19.8%), and thyroid disorders (17.3%). A total of 81.5% of patients received antihistamines and 22.8% were on omalizumab.

Table 2 shows the distribution of the scores of CU-Q_2_oL overall and dimensions’ scores, as well as reliability indicators.

**Table 2:** Distribution and reliability scores for CU-Q_2_oL

CU-Q_2_oL	# of items	Mean ± sd	Floor effect	Ceiling effect	Internal consistency α	1-week Test-retest ICC
Overall score	23	25.4 ± 19.7	4.3	0.0	0.947	0.910
Pruritus	2	40.6 ± 28.8	15.4	5.6	0.829	0.818
Swelling	2	15.6 ± 22.8	0.0	1.3	0.796	0.711
Impact on life activities	6	18.3 ± 20.7	27.2	0.6	0.869	0.862
Sleep problems	5	33.4 ± 25.5	11.9	0.6	0.899	0.866
Limits	3	27.5 ± 23.9	18.8	1.9	0.661	0.929
Looks	5	22.6 ± 24.2	23.1	0.6	0.842	0.957

*sd* standard deviation

As presented in this table, pruritus has the highest score and no CU-Q_2_oL dimension showed ceiling effect. However, some dimensions showed important floor effect (e.g., limits and looks), possibly justified by taking into account the sample characteristics. Internal consistency of the overall score (H_1_) was very good (0.947), with a small exception for the dimension ‘limits’ (0.661), and ICC showed a high reproducibility power, with a ICC for the overall score equal to 0.910, ranging from 0.711 (swelling) and 0.957 (looks) across dimensions.

### Validity

Starting by the construct validity, in our case, to test the structural validity, we opt by performing the exploratory factor analysis with all the 23 items of the Cu-Q_2_oL. Using a principal component analysis with Varimax rotation with Kaiser normalization, we selected five factors, corresponding to 73.2% of variance explained. Table 3 presents the major results from this factor analysis.

**Table 3:** Results from exploratory factor analysis on CU-Q_2_oL data

Domain	Eigen value	Items	Factor 1	Factor 2	Factor 3	Factor 4	Factor 5
Sleep problems	10.804	walking up during the night	**,834**	,143	,068	,143	,087
		daytime tiredness	**,828**	,214	,101	,213	,172
		ease of falling asleep	**,807**	,118	,271	,063	,129
		ability to concentrate	**,620**	,401	,208	,141	,266
		nervousness	**,608**	,138	,520	,092	,254
		quality of sleep	**,536**	,439	,141	,501	,023
		bad mood	**,444**	,214	,441	,119	,362
Impact on life activities	1.998	physical activities	,195	**,837**	,207	,091	,244
		use of cosmetics	,154	**,758**	,410	-,094	,224
		free time	,307	**,734**	,056	,345	,212
		social relations	,233	**,682**	,247	,344	,182
		work	,176	**,578**	,298	,474	,071
Looks	1.645	sport	,217	,159	**,807**	,295	,030
		medication adverse effects	,164	,277	**,779**	,193	,154
		embarrassing in public	,201	,318	**,707**	,085	,269
		bothersome	,101	,134	**,576**	-,031	,503
Pruritus & Swelling	1.308	lips swelling	,015	,050	,014	**,836**	,210
		eyes swelling	,133	,001	,174	**,750**	,323
		wheals	,232	,312	,134	**,732**	-,048
		pruritus	,438	,316	,299	**,583**	-,057
Limits	1.076	limits in choosing food	,198	,219	,229	,198	**,784**
		eating	,234	,301	,135	,306	**,712**
		choice of clothes	,192	,422	,286	,060	**,464**

Looking at the contents of these factors, we observed that the original two factors ‘pruritus’ and ‘swelling’ appeared merged in one sole factor, and ‘sleep problems’ original factor maintains in this new structure. Regarding the ‘impact on life activities’ factor, two items did not show together with the original ones, but with an acceptable rationale. They were the item 7 (quality of sleep) which followed the other items of the ‘sleep’ factor, as well as item 16 (bad mood), and the item 10 (eating) that appeared in the new factor ‘limits’ together with the item 17 (limits in choosing food) and the item 23 (choice of clothes). The remaining items formed the ‘looks’ factor also with item 18 (sport). At last, item 22 (use of cosmetics) appeared in the domain ‘impact on life activities’ instead of in the domain ‘looks’ (H_3_).

Another way to test the construct validity is to address the discriminative validations by looking at sociodemographic and clinical variables. Table 4 shows the different CU-Q_2_oL average scores for different sociodemographic and clinical variables.

**Table 4:** QoL perception for different levels of sociodemographic and clinical variables

		Overall score	Pruritus	Swelling	Activities	Sleep	Limits	Looks
Gender	Female	27.6	42.5	17.8	19.6	35.5	29.7	25.5
	Male	16.8	33.6	8.2	13.8	22.9	18.1	10.5
	t	3.4	1.5	2.5	1.4	2.4	2.4	4.4
	Sig	0.001	0.136	0.013	0.180	0.016	0.019	< 0.001
Age (years)	< 35	26.0	44.2	15.0	21.2	29.7	26.5	25.2
	25–44	24.6	38.4	16.3	16.3	33.4	28.6	20.1
	≥ 45	25.3	39.4	15.3	17.4	34.9	27.0	22.2
	F	0.1	0.5	0.1	0.70	0.6	0.1	0.5
	Sig	0.943	0.586	0.960	0.496	0.562	0.906	0.611
Years of education	0–4	16.6	31.9	8.3	9.9	24.6	17.8	12.0
	5–9	28.2	41.7	20.0	19.9	37.5	31.9	24.1
	10–12	25.2	43.7	14.6	19.1	32.5	27.3	20.9
	> 12	25.6	38.8	14.4	19.2	31.6	26.3	26.2
	F	1.6	0.8	1.4	1.1	1.2	1.7	1.6
	Sig	0.196	0.517	0.230	0.335	0.297	0.176	0.190
Severity of pruritus	Absent	10.4	10.4	5.4	4.9	13.5	14.7	13.5
	Mild	18.8	29.2	8.0	10.9	29.6	21.0	17.3
	Moderate	33.3	57.2	23.1	27.1	42.5	32.8	26.5
	Severe	43.9	74.4	32.7	36.3	48.6	48.3	37.1
	F	24.3	64.6	11.5	20.0	14.5	12.9	5.7
	Sig	< 0.001	< 0.001	< 0.001	< 0.001	< 0.001	< 0.001	0.001
Severity of wheals	Absent	13.7	21.0	6.7	6.3	22.5	15.8	13.4
	Mild	26.84	41.5	13.7	18.0	37.7	28.7	24.3
	Moderate	36.7	60.2	27.3	32.2	39.0	38.5	32.6
	Severe	40.8	65.0	26.2	33.7	53.0	49.2	28.5
	F	15.3	22.8	7.4	17.2	6.6	11.2	5.1
	Sig	< 0.001	< 0.001	< 0.001	< 0.001	< 0.001	< 0.001	0.002
Angioedema	Yes	30.1	46.7	25.4	22.6	35.1	32.7	27.5
	No	20.4	33.1	7.4	13.1	30.3	23.2	18.0
	t	3.0	2.9	4.8	2.7	1.1	2.4	2.4
	Sig	0.003	0.004	< 0.001	< 0.001	0.259	0.017	0.018
Urticaria control	Poor	29.1	48.1	18.4	21.7	38.0	30.4	25.5
	Well	7.7	5.3	2.7	2.2	11.9	13.9	8.9
	t	10.1	14.6	6.1	9.3	7.7	4.6	5.2
	Sig	< 0.001	< 0.001	< 0.001	< 0.001	< 0.001	< 0.001	< 0.001

t: Student’s t (2 alternatives); F: Fisher’s F (3 or more alternatives)

Regarding the sociodemographic variables and analyzing hypothesis H_4_, in general, CU-Q_2_oL differentiated well between males and females, with females always having higher QoL impairment. However, it was not able to discriminate based on age or education.

On the other hand, in what concerns clinical variables and hypothesis H_5_, this measurement instrument also differentiated well between different levels of severity of pruritus and wheals, with most severe cases scored as poor health. In addition, the presence of angioedema and a poor urticarial control were perceived as higher QoL impairment.

At last, Table 5 presents the correlations between CU-Q_2_oL overall and dimensions scores and the measurement of health status (SF-36), quality of life (EQ-5D-5 L), and a dermatology-specific questionnaire (DLQI).

**Table 5:** Correlation between QoL perception and the measures SF-26, EQ-5D-5 L and DLQI

			Total	Pruritus	Swelling	Activities	Sleep	Limits	Looks
SF-36	Physical function	ρ Sig	− 0.583 < 0.001	− 0.333 < 0.001	− 0.238 < 0.001	− 0.582 < 0.001	− 0.443 < 0.001	− 0.508 < 0.001	− 0.543 < 0.001
	Role physical	ρ Sig	−0.573 < 0.001	− 0.287 < 0.001	− 0.148 0.049	− 0.564 < 0.001	− 0.479 < 0.001	− 0.536 < 0.001	− 0.535 < 0.001
	Pain	ρ Sig	− 0.431 < 0.001	− 0.317 < 0.001	− 0.260 < 0.001	− 0.377 < 0.001	− 0.449 < 0.001	− 0.321 < 0.001	− 0.301 < 0.001
	General Health	ρ Sig	− 0.570 < 0.001	− 0.391 < 0.001	− 0.273 < 0.001	− 0.552 < 0.001	− 0.467 < 0.001	− 0.504 < 0.001	− 0.461 < 0.001
	Vitality	ρ Sig	−0.584 < 0.001	− 0.325 < 0.001	− 0.237 0.001	−0.458 < 0.001	−0.595 < 0.001	−0.584 < 0.001	−0.488 < 0.001
	Social function	ρ Sig	−0.660 < 0.001	− 0.361 < 0.001	− 0.296 < 0.001	−0.611 < 0.001	− 0.528 < 0.001	−0.658 < 0.001	− 0.591 < 0.001
	Role emotional	ρ Sig	−0.583 < 0.001	− 0.307 < 0.001	− 0.245 0.001	−0.546 < 0.001	− 0.519 < 0.001	−0.582 < 0.001	− 0.483 < 0.001
	Mental health	ρ Sig	−0.622 < 0.001	− 0.320 < 0.001	− 0.299 < 0.001	−0.474 < 0.001	− 0.625 < 0.001	−0.683 < 0.001	− 0.503 < 0.001
	Physical Summary	ρ Sig	−0.495 < 0.001	−0.337 < 0.001	− 0.217 0.004	−0.514 < 0.001	− 0.410 < 0.001	−0.373 < 0.001	− 0.417 < 0.001
	Mental Summary	ρ Sig	−0.556 < 0.001	− 0.286 < 0.001	− 0.251 0.001	−0.434 < 0.001	− 0.526 < 0.001	−0.633 < 0.001	− 0.482 < 0.001
EQ-5D-5 L	Index	ρ Sig	−0.646 < 0.001	− 0.356 < 0.001	− 0.356 < 0.001	−0.691 < 0.001	− 0.526 < 0.001	−0.532 < 0.001	− 0.504 < 0.001
	VAS	ρ Sig	−0.623 < 0.001	− 0.493 < 0.001	− 0.318 < 0.001	−0.646 < 0.001	− 0.485 < 0.001	−0.516 < 0.001	− 0.474 < 0.001
DLQI		ρ Sig	0.846 < 0.001	0.716 < 0.001	0.467 < 0.001	0.873 < 0.001	0.615 < 0.001	0.680 < 0.001	0.667 < 0.001

*ρ*: Pearson correlation coefficient; Sig: *p*-value

As expected, looking at the correlations between CU-Q_2_oL and SF-36 dimensions (hypothesis H_6_), we notice moderate negative correlations, especially for the overall CU-Q_2_oL scores, for the ‘impact on life activities’ and ‘limits’ dimensions (SF-36 physical dimensions) and for ‘sleep problems’ dimension (SF-36 mental dimensions). In addition, when CU-Q_2_oL dimensions are correlated with both EQ-5D-5 L index and VAS (hypothesis H_7_), we showed moderate and large correlation, especially with the overall CU-Q_2_oL and with ‘impact on life activities’ dimension. At last, all CU-Q_2_oL dimensions are highly correlated with DLQI index (H_8_).

## Discussion

Cu-Q_2_oL is the first disease-specific measurement instrument to address the impact of chronic urticaria on QoL. To create the Portuguese version we have followed strict methodologies based on forward-backward translations, with content, construct and criterion validity, as well as reliability tests.

The sample used to validate this version was formed by 162 chronic urticaria patients from seven centers dealing with urticaria patients from the main regions of Portugal, assuring good country coverage. Among them, 23 patients participated in a test stability over time. The sample with a mean age of 42.6 and female predominance reflects the characteristics of the population attending the chronic urticaria clinics, including in Portugal [34, 35]. All patients considered the Portuguese version understandable and without ambiguity.

Excellent reliability scores were found when performing the internal consistency and when over time stability was tested, even a little bit better than in other countries [15, 16, 20–23]. Some variability may be accepted due to the frequent changing of the disease activity over days/weeks and, consequently, with some variability in the interference on some aspects of the QoL.

Exploratory factor analysis revealed a very similar structure comparable with the one presented by the authors on its original version and explaining 73.2% of the variance. The major discrepancy between the Italian and the Portuguese CU-Q_2_oL factor structures resides on the fact that ‘pruritus’ and ‘swelling’ domains did not appear as two individual domains, encompassing a ‘symptoms’ domain. However, when comparing the structure proposed by the original authors and by the various countries’ culturally adapted versions, we also evidence some differences. In fact, while the Spanish [21] and the Turkish [23] versions retain the original scales, the German [16], the Greek [17], the Hebrew [18] and the Polish [20] versions show new six-scale structures, including dimensions as ‘functioning’ and ‘mental status’. Brazilian [15] version determined a three-scale structure formed by ‘sleep/mental status/eating’, ‘pruritus/impact on life activities’, and ‘swelling/limits/look’.”

Construct validity known-groups tests also revealed the power of CU-Q_2_oL to be able to discriminate patients based on sociodemographic, namely with a higher impact on QoL in the female population which is usually described in other studies [1, 15, 16, 20, 22, 23], and certainly has to do with higher levels of pruritus and angioedema reported in this group of females (respectively 42.5 and 17.8, compared to 33.6 and 8.2 in males).

The item ‘looks’, encompasses particularly embarrassing situations in public, use of cosmetics and choice of clothes, has shown to have a more significant impact on women and may have contributed significantly for the difference of the burden of CU between genders. In addition, when evaluated by another instrument, the DLQI, the impact of chronic spontaneous urticaria (CSU), as well as psoriasis and other chronic skin diseases is also significantly higher in females.

Clinical variables associated with more severe disease were clearly correlated with a higher score in CU-Q_2_oL. Severity of pruritus and the number of wheals in the previous week, which together constitute one of the scores more frequently used to asses disease severity in Chronic Spontaneous Urticaria (CSU) (the UAS7 – urticaria activity score 7), as well as angioedema with unpredictable swellings that often occurs in exposed areas and that may affect functional activities of patient and his life within the society (speech, visual capacity, eating, walking or manual tasks) were very significantly correlated with CU-Q_2_oL, as we might expect. Also, within the same sense, the study showed very good correlation between the score of the Cu-Q_2_oL and the UCT, that addresses questions like how severe were the symptoms and signs of CU (pruritus, wheals and swellings), how CU has interfered with the patient’s life, how much the treatment was able/unable to control the symptoms of CU, although UCT goes back to the previous four weeks [29].

The lack of effect of age and education on patients’ answers make us ensure that this measurement instrument may be used irrespective of these sociodemographic variables and that the burden of CSU is transversal to all ages and levels of education.

Comparisons between CU-Q_2_oL with the scores from DLQI, SF-36 and EQ-5D-5 L in the same population of Portuguese patients showed expected results with very good correlations between similar aspects evaluated by these different PROs further strengthening the validity of the measure obtained by the Portuguese version CU-Q_2_oL.

Also, in this study we could confirm that Portuguese results with the CU-Q_2_oL were in line with the results obtained by the original and the different versions translated in different languages and used in different populations [15–23], therefore confirming that the burden of CSU and its detrimental effect on the patients’ QoL is transversal to all populations of the world were these studies have been performed.

The possible limitation we may have in this study is the sample size. Therefore, we plan to pursue the implementation of the Portuguese version of the CU-Q_2_oL in regular medical appointments and, later, with a larger sample, to perform a confirmatory factor analysis to test the replication of the major findings.

## Conclusion

Our study showed that the Portuguese version of the CU-Q_2_oL is semantically and culturally equivalent to the original Italian version. The good performance of the scale adapted into Portuguese, its short administration time and highly cost-effective administration make the Cu-Q_2_oL a valid, reliable and useful tool for research and standard clinical practice.

## Data Availability

The data that support the findings of this study are available from the corresponding author upon reasonable request.

## Abbreviations

CSU: Chronic Spontaneous Urticaria
CU: Chronic Urticaria
CU-Q2oL: Chronic Urticaria Quality of Life Questionnaire
DLQI: Dermatology Quality of Life Index
ICC: Intraclasse Correlation Coefficient
QoL: Quality of Life
SF-36: Short Form Health Survey
UAS7: Urticaria Activity Score 7
UCT: Urticaria Control Test
